# Designing and implementing an intelligent vaccine logistics management system for India’s Universal Immunisation Programme (UIP) - ‘The eVIN Model’

**DOI:** 10.1186/2052-3211-7-S1-O3

**Published:** 2014-12-17

**Authors:** Bhrigu Kapuria, Juthika Talukdar, Nithiyananthan Muthusamy, Rajeev Gera

**Affiliations:** 1 Immunisation Technical Support Unit (ITSU) - Public Health Foundation of India (PHFI), New Delhi, India; 2 IPE Global Private Limited, New Delhi, India

## Background

India’s full immunisation coverage for infants is 61%. The availability of quality vaccines at session sites is a contributor to low coverage. Weaknesses in the current supply chain include lack of stock visibility, poor distribution planning, and improper storage conditions. A national vaccine logistic management system is required which provides visibility of real time stock levels across all cold chain points, and enables staff to apply logistics management principles for vaccines.

## Method

With the objective of identifying weaknesses, and their root causes, in India’s vaccine logistics system, ITSU conducted a ‘Deep Dive Study’ in three states. ITSU then commissioned a feasibility study on involving private sector players to address identified gaps. ITSU used these findings to design and pilot the electronic Vaccine Intelligence Network (eVIN), which is comprised of trained Vaccine and Cold Chain Managers (VCCMs) integrated into a supportive supervision approach, user-friendly technology, and standardized processes.

## Results

eVIN is currently being piloted in two geographical locations catering to a population of 7.4 million, with one VCCM in each location. eVIN’s impact is being assessed and early results indicate high levels of system adoption by cold chain staff, and high stock data quality, driven by HR strengthening measures.

eVIN has been adopted by the Ministry of Health, Government of India for the National Immunization Programme. The VCCM cadre is now being scaled in 3 major Indian states with a combined population of 345 million. The introduction of the VCCM cadre at the all-India level is currently being considered.

## Discussion

The core of an effective vaccine logistics management system is a well-supported operations team that uses technology platforms to make intelligent distribution decisions. Hence, human resources is an integral component of any conceptual design and implementation plan for a vaccine logistics intervention. Focusing solely on technology solutions will have a limited effect in public health programmes especially in resource-poor settings. User-friendly technology, when married to additional human resources, a defined supportive supervision plan and a rigorous training regimen for existing staff results in high adoption rates and high data quality, as evidenced by the eVIN pilot thus far.

## Lessons learned

While designing eVIN, and piloting it thus far, it was learned that any vaccine logistics system which aims for sustainable performance and health systems strengthening in resource poor settings needs to adequately map the required workload, define measures to augment human resources, rigorously support existing staff, and define clear processes.

